# Quantitative SSTR-PET/CT for predicting response and survival outcomes in patients with pancreatic neuroendocrine tumors receiving CAPTEM

**DOI:** 10.2478/raon-2023-0055

**Published:** 2023-11-30

**Authors:** Maria Ingenerf, Homeira Karim, Christoph Auernhammer, Matthias Zacherl, Vera Wenter, Michael Winkelmann, Jens Ricke, Frank Berger, Christine Schmid-Tannwald

**Affiliations:** Department of Radiology, University Hospital, LMU Munich, Munich, Germany; Department of Nuclear Medicine, University Hospital, LMU Munich, Munich, Germany; ENETS Centre of Excellence, Interdisciplinary Center of Neuroendocrine Tumours of the GastroEnteroPancreatic System at the University Hospital of Munich (GEPNET-KUM), University Hospital of Munich, Munich, Germany; Department of Internal Medicine, University Hospital, LMU Munich, Munich, Germany

**Keywords:** prognosis, positron emission tomography–computed tomography, neuroendocrine tumors, capecitabine/temozolomide

## Abstract

**Background:**

This study aimed to evaluate the predictive and monitoring role of somatostatin receptor (SSTR) positron emission tomography-computed tomography (PET/CT) and clinical parameters in patients with neuroendocrine liver metastases (NELM) from pancreatic neuroendocrine tumors (pNET) receiving capecitabine and temozolomide (CAPTEM).

**Patients and methods:**

This retrospective study included twenty-two patients with pNET and NELM receiving CAPTEM who underwent pre- and post-therapeutic ^68^Ga-DOTATATE/-TOC PET/CT. Imaging (including standardized uptake value [SUV] of target lesions [NELM and pNET], normal spleen and liver) and clinical (Chromogranin A [CgA], Ki-67) parameters were assessed. Treatment outcome was evaluated as response according to RECIST 1.1, progression free survival (PFS) and overall survival (OS).

**Results:**

The median PFS (mPFS) was 7 months. Responders had a significantly longer mPFS compared to non-responders (10 *vs*. 4 months p = 0.022). Median OS (mOS) was 33 months (mOS: responders = 80 months, non-responders = 24 months p = 0.182). Baseline imaging showed higher SUV in responders, including absolute SUV, tumor-to-spleen (T/S), and tumor-to-liver (T/L) ratios (p < 0.02). All SUV parameters changed only in the responders during follow-up. Univariable Cox regression analysis identified baseline Tmax/Smean ratio and percentage change in size of pNETs as significant factors associated with PFS. A baseline Tmax/Smean ratio < 1.5 was associated with a shorter mPFS (10 *vs*. 4 months, (p < 0.05)). Prognostic factors for OS included age, percentage change in CgA and in T/S ratios in univariable Cox regression.

**Conclusions:**

SSTR-PET/CT can be useful for predicting response and survival outcomes in pNET patients receiving CAPTEM: Higher baseline SUV values, particularly Tmax/Smean ratios of liver metastases were associated with better response and prolonged PFS.

## Introduction

Capecitabine/temozolomide (CAPTEM) has shown to be effective and safe in advanced NETs, particularly in well-differentiated pNETs.^1,2^ CAPTEM is included in national and international guidelines for the treatment of gastroenteropancreatic neuroendocrine neoplasms, such as those by the European Society for Medical Oncology (ESMO).^3,4^ In vitro studies have demonstrated an apoptotic synergism between capecitabine and temozolomide (CAPTEM), although the exact mechanism of action in NETs remains unclear.^3,5^ Capecitabine incorporates 5-fluorodeoxyuridine triphosphate into DNA, inhibiting thymidylate synthase and attenuating the repair activity of methylguanine DNA methyltransferase (MGMT).^3^ Temozolomide exerts a cytotoxic effect through DNA alkylation/methylation at the O6 and N7 positions of guanine, leading to DNA mismatch and tumor cell death.^6^

To improve the selection of patients who would benefit from this cytotoxic regimen and avoid unnecessary toxicity due to treatment failure, predictive biomarkers need to be identified.^7^ Potential predictive biomarkers, such as MGMT expression, tumor grade and serum alanine aminotransferase (ALT) activation, have been investigated; however, the results have been controversial. A study by Cives *et al*. did not recommend biomarker-driven selection criteria for the use of the CAPTEM regimen.^6^ Conversely, Wang *et al*. identified the Ki-67 index as the only independent prognostic factor for overall survival and PFS.^7^

In addition to the size based RECIST 1.1. criteria, other imaging parameters are increasingly being evaluated for predicting and monitoring oncologic therapy concepts. The Choi criteria, which integrate changes in tumor density show a better correlation with OS than RECIST in the therapy evaluation of pNET under sunitinib.^8^ This may be attributed to antiproliferative or antiangiogenic effects, particularly in slow-growing tumors such as NETs.^9,10,11^

Approximately 80–95% of good to moderately differentiated NETs overexpress somatostatin receptors (SSTRs) on cell surfaces. PET/CT with ^68^Ga-labeled somatostatin analogues (SSA) (^68^Ga-DOTA-TATE, -DOTA-NOC and -DOTA-TOC) allows visualization of SSTRs and correlates with the histopathological expression of SSTRs.^12,13,14^ SSTR-PET/CT enables detection of NET and its metastases with high sensitivity and specificity^15^ and it is recommended for initial staging and follow-up of gastroenteropancreatic neuroendocrine tumors (GEP-NET) by the European Society for Medical Oncology Guidelines Working Group.^4^

Although quantitative evaluation of SSTR imaging has not yet been standardized, several studies suggest that these tracers could serve as parameters for therapy monitoring and response prediction in various therapeutic approaches in NET patients^16^ including those undergoing peptide-receptor-radionulcide therapy (PRRT)^17^, or transarterial radioembolization (TARE).^18,19^

However, to date, no study has investigated the role of SSTR-PET/CT parameters in predicting and assessing tumor response in patients with pNET with liver metastases treated with CAPTEM. Therefore, the aim of this study was to evaluate morphologic and functional imaging factors for predicting and monitoring the therapy response in patients with metastatic pNET treated with CAPTEM.

## Patients and methods

### Patients

This retrospective study included consecutive patients with histologically proven pNETs who received CAPTEM treatment and underwent pre- and post-therapeutic ^68^Ga-DOTA-TATE or -DOTA-TOC PET/CT imaging at our department with therapy start between 2012 and 2020. Patient selection for CAPTEM therapy was based on consensus decisions made in an interdisciplinary tumor conference certified for NETs at our ENETS Center of Excellence. The study was approved by the local research ethics committee (#20–1077), and written informed consent was waived due to the retrospective nature of the study. All procedures performed in studies involving human participants were in accordance with the 1964 Helsinki declaration and its later amendments or comparable ethical standards.

### PET/CT

Whole-body PET scans were conducted using either a GE Discovery 690 (GE Healthcare, Little Chalfont, United Kingdom) or a Biograph 64 TruePoint PET/CT scanner (Siemens Healthcare, Erlangen, Germany) in 3-D mode with a duration of 3 minutes per bed position. The emission sequence began 60 minutes after intravenous administration of approximately 200 MBq of ^68^Ga-DOTA-TATE or ^68^Ga-DOTA-TOC, along with a possible administration of 20 mg of furosemide. Emission data were reconstructed with attenuation correction based on a diagnostic CT scan.

PET/CT scans encompassed the neck, thorax, abdomen, and pelvis, utilizing a diagnostic CT scan protocol (100–190 mAs, 120 kV, collimation 2 × 5 mm, pitch of 1.5). An iodine-based contrast agent (Ultravist 300TM; Bayer Healthcare, Berlin, Germany; 1.5 mL/kg body weight) was intravenously injected at a rate of 2.5 mL/s with a delay of 80–110 seconds to visualize the portal venous phase of the liver. PET images were reconstructed using specific parameters: a transaxial 256 × 256 matrix with VPFX (2 iterations, 36 subsets, 3D Gauss postfilter of 6.5 mm full-width half maximum) for the GE scanner and a transaxial 168 × 168 matrix with TrueX (3 iterations, 21 subsets, 3D Gauss postfilter of 2.0 mm full-width half maximum) for the Biograph scanner. Standardized uptake values (SUV) were calculated using the patient's body weight (SUVbw).

### Image analysis

PET/CT scans were reviewed by two board-certified radiologists in consensus, who were blinded to the clinical history of the patients, except for the diagnosis of pNET. Two target neuroendocrine liver metastases (NELM) larger than 1 cm in size per patient were defined as target lesions based on visibility on CT scan and visual positive somatostatin receptor (SSTR) uptake compared to normal liver parenchyma, with no artifacts within the lesions.

The image review process consisted of two separate sessions conducted at a 3-week interval (1. analysis of pre-therapeutic PET/CT and 2. analysis of post-therapeutic PET/CT). In each session, the size and density (measured in HU) of the selected liver metastases and pNET were recorded. Additionally, the HU of the normal liver parenchyma and spleen were measured. The hepatic tumor burden was assessed visually. Semi-quantitative measurements were performed by placing circular volume of interest (VOI) in the respective target lesions/organs to obtain maximum and mean standardized uptake values (SUVs) of the liver metastases, pNET, normal liver parenchyma, and healthy spleen parenchyma. SUV tumor-to-organ ratios, including tumor-to-spleen (T/S) ratio and tumor-to-liver (T/L) ratio, were calculated as SUVmax (liver metastasis) divided by SUVmean (normal liver or spleen respectively) and SUVmean (liver metastasis) divided by SUVmean (liver or spleen) for normalization purposes.

### Standard of reference and response to treatment

Clinical, histopathological, surgical records of each patient were collected by a third radiologist. The histopathological diagnosis of pNET and the Ki-67 labeling index of the primary tumor were confirmed for all patients. Tumor grading was performed based on WHO Tumor Classification Guideline, which categorized tumors into G1 (Ki-67 Index < 3%), G2 (Ki-67 Index 3–20%), and G3 NET/NEC (Ki-67 Index > 20%).

The treatment response was assessed using RECIST 1.1 criteria. Patients were classified as responders (R) if they achieved a complete response (CR) or partial response (PR) based on the first follow-up PET/CT scan. Non-responders were defined as patients with stable disease (SD) or progressive disease (PD). Progression-free survival (PFS) was calculated in months from the initiation of CAPTEM treatment until progression, as determined by imaging and clinical parameters according to the local interdisciplinary tumor board. Overall survival (OS) was measured in months from the start of CAPTEM treatment until death from any cause. Patients who were still alive at the last follow-up in December 2022 were censored.

### Statistical analysis

All data were presented as mean or median values with standard deviation (SD) or interquartile range [IQR], respectively. The normal distribution of continuous variables was assessed by visually inspecting the frequency distribution using histograms.

To compare imaging parameters such as SUV, SUV tumor-to-spleen (T/S) ratios, SUV tumor-to-liver (T/L) ratios, size, HU, and quantitative clinical parameters before and after therapy, a Wilcoxon signed rank test was employed. The Mann-Whitney test was used to compare these parameters between different response groups.

OS and PFS were analysed using the Kaplan-Meier method, and survival curves were compared using the Breslow-Wilcoxon test. Prognostic clinical and imaging parameters for PFS and OS were analysed using Cox proportional hazards regression. In the multivariable model, variables with a p-value ≤ 0.05 in the univariable analysis were included using a stepwise approach. A statistical significance level of p ≤ 0.05 was considered significant. Statistical analyses were conducted using commercially available software, including GraphPad Prism Version 6 (San Diego, CA) and SPSS version 25 (Chicago, IL).

## Results

### Patients characteristics

22 patients with a total of 44 target NELM and 16 pNETs were included for PET/CT analysis. For density measurements, two patients were disregarded due to non-contrast-enhanced CT scans. Six patients underwent surgery with resection of pNET and three patients underwent splenectomy. The baseline PET/CT scans were obtained 58 d prior to CAPTEM initiation (IQR 32 –103d), and follow-up PET/CT scans were performed 132 d after the start of therapy (IQR 88 – 192d). The majority of patients were male (77%), and most had G2 tumors (77%), with a minority having G3 tumors (18%). Detailed patient characteristics are presented in Table 1.

**TABLE 1.** Patient characteristics

**Sex**	
Male	17 (77%)
Female	5 (23%)
Median age, years (range)	66 (40–85)
**Grading**	
G1	1 (5%)
G2	17 (77%)
G3	4 (18%)
Median Ki-67 (ng/ml, range)	12 (2–40)
**Treatment with CAPTEM**	
Duration of treatment (month, range)	7.5 (3–20)
Prior treatment	16 (73%)
pNET resected	6 (27%)
Prior medical treatment	9 (41%)
Prior PRRT	6 (27%)
Prior liver targeted therapy	6 (27%)

CAPTEM = capecitabine and temozolomide; pNET = pancreatic neuroendocrine tumors; PRRT = peptide-receptor-radionulcide therapy

### OS, PFS and Treatment response according to RECIST 1.1

According to RECIST 1.1 criteria, 10 patients exhibited a partial response (PR), 11 patients had stable disease (SD), and 1 patient showed progressive disease (PD) on the first follow-up PET/CT scan. The objective response rate (ORR) was 46%, with PD and SD classified as non-responders.

The overall median PFS was 7 months (95% CI: 1.5–12.5 months). Among the responders (n = 10), the median PFS was 10 months (95% CI: 6.9–13.1 months), while in the non-responder group (n = 12), it was 4 months (95% CI: 3.3–4.7 months). The difference in PFS between the two groups was statistically significant (p = 0.022).

The overall median OS was 33 months (95% CI: 0–77 months). Among the responders (n = 10), the median OS was 80 months (95% CI: 11–149 months), whereas in the non-responder group (n = 12), it was 24 months (95% CI: 14–34 months). Although the difference in OS between the two groups was not statistically significant (p = 0.182).

### Response according to RECIST 1.1.

#### Prognostic imaging and clinical parameters

Significant differences were observed in the response groups based on RECIST 1.1 criteria. Responding NELM exhibited higher baseline SUVmax (47 *vs*. 24, p < 0.02) and higher baseline SUVmean (30 *vs*. 14, p < 0.004) compared to non-responding lesions (Table 2). Furthermore, the SUV ratios of Tumor/Liver (T/L ratio) and Tumor/Spleen (T/S ratio) were significantly different between the response groups on pretreatment imaging, with responding NELM demonstrating higher ratios. For instance, the Tmean/Lmean ratio was 4.8 (R) compared to 2.1 (NR) (p = 0.006). However, there were no significant differences in SUVmax and SUVmean of the primary neuroendocrine tumor (pNET) between the response groups. Baseline clinical and laboratory parameters did not differ between the response groups (Table 2).

**TABLE 2.** Imaging and clinical parameters on baseline and follow-up imaging

	**Responder**			**Non-Responder**			**Baseline R *vs*. NR**

	**Baseline**	**Follow-up**	**p-value baseline *vs.* FU**	**Baseline**	**Follow-up**	**p-value baseline *vs.* FU**	**p-value**
**Age (years)**	66 (56–75)			69 (57–82)			0.44
**Grading**	13 (7–20)			10 (4–15)			0.33
** G1**	0			1			
** G2**	6			11			
** G3**	4			0			
**Ki-67 (%)**	12.5 (7.3–20)			10 (4.3–15)			0.33
**Male sex**	7 (70%)			10 (83%)			
**Chromogranin A**	796 (512–2756)	270 (102–1136)	**< 0.04**	178 (90–845)	198 (96–1071)	**< 0.02**	0.06
**Bilirubin (mg/dl)**	0.6 (0.60.88)	0.85 (0.5–1.1)	0.64	0.6 (0.43–0.9)	0.55 (0.33–0.88)	> 0.99	0.45
**Hepatic tumor burden (%)**	35 (5–40)	13 (5–33)	0.16	13 (5–38)	15 (5–38)	0.25	0.42
**SUVmax LM**	47 (24–62)	21 (13–46)	**< 0.04**	24 (13–43)	21 (11–39)	0.15	**< 0.02**
**SUVmean LM**	30 (15–38)	15 (11–24)	**0.04**	14 (9–22)	11 (8–17)	0.40	**< 0.0004**
**Tmax/Lmean**	6.9 (3.2–11.3)	2.8 (1.6–8.3)	**0.03**	3.6 (2.0–5.8)	3.2 (1.5–5.5)	0.6	**0.0192**
**Tmean/Lmean**	4.8 (2.0–6.8)	1.9 (1.2–3.9)	**0.02**	2.1 (1.4–2.9)	1.6 (1.1–2.6)	**0.05**	**0.0061**
**Tmax/Smean**	2.0 (1.4–4.1)	0.9 (0.5–3.0)	**0.01**	1.3 (0.5–1.9)	1.3 (0.4–2.2)	0.12	**0.0469**
**Tmean/Smean**	1.4 (0.8–2.1)	0.7 (0.4–1.5)	**0.007**	0.7 (0.4–1.1)	0.6 (0.3–1.1)	0.19	**0.0094**
**Size LM (mm)**	32 (24–42)	20 (14–32)	**< 0.0001**	27 (17–36)	30 (18–43)	0.12	0.10
**HU LM**	106 (88–116)	106 (95–126)	0.41	92 (85–108)	90 (68 –104)	**0.02**	0.09
**SUVmax pNET**	26 (14–47)	26 (16–41)	0.94	30 (12–59)	28 (16–45)	0.2	0.68
**SUVmean pNET**	17 (9–22)	18 (13–28)	0.81	15 (9–33)	15 (15–30)	0.57	0.98
**Size pNET**	35 (25–38)	20 (14–37)	**0.03**	34 (20–47)	34 (23–54)	0.81	0.92
**HU pNET**	105 (77–113)	94 (88–98)	0.79	107 (81–117)	92 (81–100)	0.15	0.89

FU = Follow-up; HU = Hounsfied unit, L = liver; LM = liver metastases; pNET = pancreatic neuroendocrine tumor; SUV = standardized uptake value; S = spleen; T = tumor

Data are given as median (25^th^ and 75^th^ percentile) or number (percentage)

#### Changes between baseline and follow-up imaging

**FIGURE 1.**
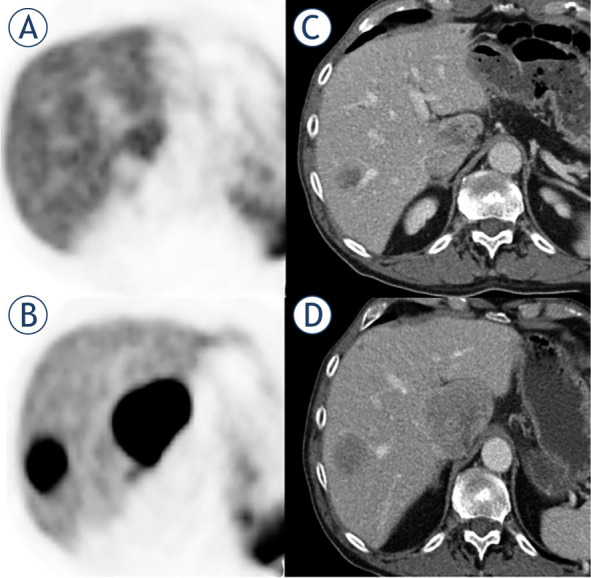
72-year-old male with responding liver metastases of pancreatic neuroendocrine tumor. on the pretherapeutic PET/CT **(A, B)** there were high tumor-to-liver (t/l) ratios. after three months of treatment with CAPTEM, both liver metastases showed a shrinkage in size, but also a significantly reduced uptake of ^68^ga-dotatate compared to pretherapeutic PET/ CT **(C, D)**. CAPTEM = capecitabine and temozolomide

Significant changes were observed in SUVmax and SUVmean of the liver metastasis between baseline and follow-up imaging in responders, whereas no changes were observed in non-responders (Figure 1). Similarly, the T/L and T/S ratios showed a significant decrease in responders, while there was no relevant change in non-responders, except for the Tmean/Lmean ratio, which also demonstrated a decrease in non-responder. The HU of the NELM exhibited a slightly significant decrease in non-responder (p = 0.02), while no significant change was observed in responding lesions. In terms of clinical parameters, CgA demonstrated a significant decrease of 72% in responders, whereas there was a slight increase of 41% non-responders (Table 2).

### Cox regression analysis for progression free survival (PFS)

In the univariable analysis, both the baseline Tmax/Smean ratio and the percentage change in the size of pNETs demonstrated a significant association with PFS (Table 3). However, in the multivariable model, none of the parameters remained statistically significant. Nonetheless, it is worth noting that the baseline Tmax/Smean ratio showed a borderline association (p = 0.09), which may be attributed to the limited sample size. Subsequently, a receiver operating characteristic (ROC) analysis was performed, revealing a baseline Tmax/Smean ratio of < 1.5 as the optimal cutoff for identifying patients with a shorter median PFS (sensitivity: 60%, specificity: 89%), with a median PFS of 10 months compared to 4 months in the lower ratio group (p = 0.047). These results were comparable to the PFS when classified by RECIST with a median PFS of 10 months (95% CI: 6.9–13.1) in the responder (R) group (n = 10), whereas it was 4 months in the non-responder (NR) group (n = 12) (95% CI: 3.3–4.7, p = 0.022).

**TABLE 3.** Cox regression analysis for progression free survival (PFS)

	**Univariable**			**Multivariable**		

	**Exp (B)**	**95% CI**	**p-value**	**Exp (B)**	**95% CI**	**p-value**
**Baseline**						
Age (years)	1.037	0.995–1.082	0.084			
Sex	1.105	0.324–2.528	0.849			
Grading						
G1			0.251			
G2	0	0	0.985			
G3	2.913	0.827–10.625	0.096			
Ki-67	1.02	0.978–1.063	0.362			
Chromogranin	1	1	0.989			
Hepatic tumor burden	0.98	0.955–1.006	0.125			
SUVmax LM	0.979	0.951–1.007	0.134			
SUVmean LM	0.982	0.94–1.025	0.41			
SUVmax Pancreas	0.983	0.956–1.01	0.21			
SUVmean Pancreas	0.975	0.93–1.023	0.3			
Tmax/Lmean	0.922	0.821–1.037	0.175			
Tmean/Lmean	0.886	0.72–1.09	0.251			
Tmax/Smean	0.588	0.373–0.927	**0.022**	0.626	0.365–1.076	0.09
Tmean/Smean	0.474	0.223–1.004	0.051			
**% change**						
CgA	1.004	1–1.008	0.056			
SUVmax LM	1.002	0.99–1.013	0.792			
SUVmean LM	0.998	0.986.1.009	0.686			
HU LM	0.999	0.975–1.024	0.948			
SUVmax Pancreas	1.002	0.996–1.008	0.451			
SUVmean Pancreas	1.002	0.995–1.009	0.512			
Tmax/Lmean	1.003	0.992–1.013	0.623			
Tmean/Lmean	0.99	0.989–1.009	0.836			
Tmax/Smean	1.006	0.99–1.013	0.079			
Tmean/Smean	1.006	0.998–1.013	0.149			
Size LM (mm)	0.999	0.983–1.015	0.908			
Size Pancreas	1.018	1.001–1.034	**0.037**	1.009	0.99–1.009	0.37
Size total (RECIST)	1.014	0.993–1.036	0.183			

HU = Hounsfied unit; L = liver; LM = liver metastases; pNET = pancreatic neuroendocrine tumor; SUV = standardized uptake value; S = spleen; T = tumor

### Cox regression for overall survival (OS)

In the univariable analysis, patient age, percent change of CgA, percent changes of Tmax/Smean ratio, and Tmean/Smean ratio were found to be significant factors associated with survival (Table 4). However, in the multivariable analysis, none of these parameters remained significant, although patient age showed borderline significance with a p-value of 0.07. In a subsequent receiver operating characteristic (ROC) analysis, a percent change of Tmean/Smean ratio ≥ −35 was identified as the optimal cutoff for stratifying patients with a shorter median OS after treatment. This cutoff had a sensitivity of 80% and specificity of 67%. Mean OS was 71 months (95% CI: 10–53 months) compared to 44 months (95% CI: 14–17 months) in the lower percentage decrease group (p = 0.034). No significant difference in OS was observed between responder (R) and non-responder (NR) groups according to RECIST 1.1. Mean OS was 60 months (95% CI: 9–42 months) in the R group compared to 56 months (95% CI: 15–28 months) in the NR group (p = 0.182).

**TABLE 4.** Cox regression analysis for overall survival (OS)

	**Univariable**			**Multivariable**		

	**Exp (B)**	**95% CI**	**p-value**	**Exp (B)**	**95% CI**	**p-value**
**Baseline**						
Age (years)	1.059	1.003–1.118	**0.039**	1.054	0.996–1.115	0.071
Sex	2.326	0.486–11.4	0.291			
Grading						
*G1*			0.64			
*G2*	0	0	0.988			
*G3*	2.137	0.442–10.325	0.345			
Ki-67	1.033	0.979–1.09	0.24			
Chromgranin	1	1.0–1.0	0.572			
Hepatic tumor burden	1.005	0.974–1.036	0.776			
SUVmax LM	0.982	0.949–1.016	0.294			
SUVmean LM	0.951	0.894–1.012	0.114			
SUVmax Pankreas	0.962	0.916–1.011	0.125			
SUVmean Pankreas	0.912	0.813–1.023	0.115			
Tmax/Lmean	0.988	0.856–1.142	0.874			
Tmean/Lmean	0.879	0.653–1.184	0.396			
Tmax/Smean	0.83	0.516–1.336	0.443			
Tmean/Smean	0.615	0.239–1.581	0.313			
**% change**						
CgA	1.006	1.0–1.012	**0.046**	1.003	0.996–1.01	0.448
SUVmax LM	1.004	0.99–1.018	0.563			
SUVmean LM	1.006	0.99–1.022	0.493			
HU LM	0.986	0.956–1.017	0.374			
SUVmax Pankreas	1.006	0.998–1.013	0.118			
SUVmean Pankreas	1.007	0.998–1.015	0.132			
Tmax/Lmean	1	0.989–1.011	0.986			
Tmean/Lmean	1.001	0.988–1.013	0.933			
Tmax/Smean	1.014	1.002–1.026	**0.02**	1.008	0.971–1.046	0.68
Tmean/Smean	1.017	1.003–1.032	**0.02**	1.011	0.973–1.05	0.58
Size LM (mm)	1.011	0.991–1.031	0.289			
Size Pancreas	1.004	0.986–1.023	0.672			
Size total (RECIST)	1.009	0.988–1.031	0.409			

HU = Hounsfied unit; L = liver; LM = liver metastases; pNET = pancreatic neuroendocrine tumor; SUV = standardized uptake value; S = spleen; T = tumor

## Discussion

In this study we investigated the use of clinical, morphological, and functional imaging parameters for response assessment and prediction of pNETs treated by CAPTEM. Our findings highlight the potential of quantitative SSTR-PET/CT as a valuable tool for predicting and monitoring treatment response and survival in pNET patients receiving CAPTEM therapy.

The overall median PFS in our cohort was 7 months, which appears relatively low compared to the range of 6 to 34 months reported in the literature for advanced neuroendocrine neoplasms regardless tumor site of origin.^3^ However, reported PFS times vary considerably and are likely influenced by factors such as tumor grading, prior treatments, and variations in the administration of the CAPTEM regimen across studies. Our cohort consisted of rather heavily pretreated patients, which may contribute to these differences, as also observed in a retrospective work by D’Alpino Peixoto *et al*.^20^ The overall median OS in our population was 33 months, consistent with recent studies reporting median OS times ranging from 29 to 75 months.^3,21^ Generally, pNETs are known to have a better response to the CAPTEM regimen compared to non-pancreatic NETs.^20.21^

The ORR according to RECIST was rather high in our cohort, reaching 45%, while other studies focusing on pNETs reported ORRs between 21% and 54%.^3,21^ In our study, treatment response based on RECIST 1.1 showed a slightly improved PFS of 10 months compared to 4 months; however, this did not result in a significant increase in OS. It should be noted that the assessment of OS can be challenging in slow-growing tumor types like NET, where patients often have long survival times and receive a variety of different post-progression therapy regimens.

Interestingly, in our study, baseline imaging revealed significantly higher SUV in liver metastases that responded to treatment compared to non-responding lesions. Moreover, all calculated SUV ratios including tumor-to-liver (T/L) ratios and tumor-to-spleen (T/S) ratios, were significantly higher in responding lesions. We also found that a higher baseline Tmax/Smean of NELM was associated with longer PFS in our univariable Cox regression analysis (HR 0.59; 95% CI 0.37–0.93, p = 0.02). Using a cutoff of > 1.5 for baseline Tmax/Smean yielded similar median PFS times as response classification according to RECIST 1.1, with 10 months compared to 4 months.

Several studies investigating peptide-receptor-radionulcide therapy (PRRT) have also reported higher baseline SUV and pretherapeutic T/L ratios as prognostic factors for a better treatment response, suggesting cutoffs of SUVmax between 13 and 18 to distinguish responders from non-responders.^23,24,25^ This observation can be explained by the fact that PRRT is a receptor-directed treatment approach, where SUV values roughly correspond to the dose delivered by PRRT.^23^ However, the underlying mechanism in the context of cytoreductive therapy, such as CAPTEM, remains unclear. One possible explanation is that a target lesion with a higher SSTR-expression might be more differentiated, although this contrasts with the common observation that particularly high-grade NET profit from CAPTEM treatment.

On follow-up imaging, we observed significant changes in SUV parameters in NELM that responded to treatment, whereas non-responding NELM showed no changes between baseline and follow-up imaging. However, despite this finding, we did not find an association between percentage changes in SUV parameters and PFS in our regression analysis. Interestingly, we did find that percentage changes in Tmax/Smean and Tmean/Smean were significantly associated with OS in our univariable analysis (e.g., Tmean/Smean HR 1.017, 95% CI 1.003–1.032, p = 0.02), although these association did not remain significant in our multivariable analysis. A percentual decrease in the Tmean/Smean ratio ≤ −35% was associated with a slightly longer mean OS of 71 months compared to 44 months (p = 0.03).

In relation to FDG PET/CT, several studies have demonstrated that SUV reduction after treatment can predict survival. For example, this has been observed in patients with liver metastasis of pancreatic cancer treated with TARE and in breast cancer patients receiving targeted therapies.^26,27^

Regarding the primary tumor we did not detect any statistically significant changes in SUV during treatment and between response groups. Only the size of the pNETs decreased significantly in responders while there was no change in non-responders. These observations might be related to the small number of total pNET (n = 16). Another possibility is that functional / morphological changes might be different between liver metastasis and the primary tumor as discussed by Ingenerf *et al*.^28^

The Choi *et al.* response criteria suggested that changes in tumor attenuation could better represent treatment response to imatinib in gastrointestinal stromal tumors.^29^ While Choi *et al.* found a decrease in tumor density of more than 15% on CT had a sensitivity of 97% and a specificity of 100% in identifying PET responders versus 52% and 100% by RECIST, we observed a slight, but statistically significant decrease in HU values in non-responding lesions (p = 0.02), while no significant change was observed in responding lesions.

Regarding clinical parameters, changes in CgA were significantly different between response groups according to RECIST (responders: −72% *vs*. non-responders: + 41%). Also, percentage changes of CgA were identified as predictive factor for OS, although statistical significance was not reached in the multivariable model. It should be noted that CgA levels can be influenced by various conditions such as gastritis and liver cirrhosis, limiting its use as a tumor marker despite its correlation with tumor progression in several studies.^30,32^

Another potential biomarker, Ki-67, has controversial applicability in patients with neuroendocrine tumors (NETs) receiving CAPTEM therapy.^3,33,34^ Therefore, at present, no biomarker-driven selection criteria for use of the CAPTEM regimen can be recommended.^6^ In our patient cohort, we found no differences in Ki-67 between response groups, and no correlation with OS or PFS. It is important to note that the small number of patients with G1 (n = 1) and G3 (n = 4) tumors in our cohort might have limited the ability to detect significant associations.

The main limitations of this study were its small patient cohort and its retrospective nature, accompanied by heterogeneous time intervals between PET/CTs and CAPTEM initiation, as well as heterogenous prior therapies. Additionally, while the quantification of SUVs is well-established for FDG PET/CT using the PERCIST criteria^35^, its application to SSTR-PET/CT is less established. Therefore, caution must be exercised when interpreting SUV changes, as they can be attributed to tumor regression or dedifferentiation.^23^ Some authors have suggested that normalized SUV measures, such as tumor-to-spleen, liver, or blood pool ratios, may be more reliable than absolute SUV measurements.^36,37^ Another limitation is that patients underwent scans using different scanners and different somatostatin analogs, which further supports the preference for tumor-to-organ ratios. Furthermore, in some cases, the pre- and post-treatment scans from the same patient was performed on different scanners.

Our study highlights the potential value of quantitative SSTR-PET/CT in predicting response and survival outcomes in patients with pNETs receiving CAPTEM. Responders exhibited higher SUV values on baseline imaging, and the baseline Tmax/Smean ratio showed a significant association with PFS. Moreover, the observed significant decrease in SUV values in responding NELM during follow-up imaging supports the utility of these parameters for treatment monitoring. These findings provide valuable insights into non-invasive tools for guiding treatment strategies, monitoring response, and predicting patient outcomes. Further research is needed to validate and expand upon these findings. Incorporating these parameters into routine clinical practice could enhance patient care and enable more personalized treatment approaches.
